# Evaluation of Regulatory Immune Response in Skin Lesions of Patients Affected by Nonulcerated or Atypical Cutaneous Leishmaniasis in Honduras, Central America

**DOI:** 10.1155/2018/3487591

**Published:** 2018-03-21

**Authors:** Gabriela Venicia Araujo Flores, Carmen Maria Sandoval Pacheco, Thaise Yumie Tomokane, Wilfredo Sosa Ochoa, Concepción Zúniga Valeriano, Claudia Maria Castro Gomes, Carlos Eduardo Pereira Corbett, Marcia Dalastra Laurenti

**Affiliations:** ^1^Laboratory of Pathology of Infectious Diseases, Medical School, University of São Paulo, São Paulo, SP, Brazil; ^2^Microbiology School, National Autonomous University of Honduras, Tegucigalpa, Honduras; ^3^University School Hospital, Tegucigalpa, Honduras

## Abstract

In Honduras, *Leishmania (L.) infantum chagasi* causes both visceral leishmaniasis (LV) and nonulcerated or atypical cutaneous leishmaniasis (NUCL). NUCL is characterized by mononuclear inflammatory infiltration of the dermis, composed mainly of lymphocytes followed by macrophages with discrete parasitism. Considering that little is known about the pathogenesis of NUCL, the aim of this study was to evaluate the regulatory response in situ in skin lesions of patients affected by NUCL. Biopsies (*n* = 20) from human cutaneous nonulcerative lesions were collected and processed by usual histological techniques. The in situ regulatory immune response was evaluated by immunohistochemistry using antihuman CD4, FoxP3, IL-10, and TGF-*β* antibodies. CD4^+^, FoxP3^+^, TGF-*β*^+^, and IL-10^+^ cells were observed in the dermis with inflammatory infiltration in all studied cases and at higher densities compared to the normal skin controls. A positive and strong correlation was observed between CD4^+^ and FoxP3^+^ cells, and a positive and moderate correlation was observed between FoxP3^+^ and TGF-*β*^+^ but not with IL-10^+^ cells. The data suggest that T regulatory FoxP3^+^ cells and the regulatory cytokines, especially TGF-*β*, play an important role in the immunopathogenesis of NUCL, modulating a cellular immune response in the skin, avoiding tissue damage, and leading to low tissue parasitic persistence.

## 1. Introduction

Nonulcerated cutaneous leishmaniasis (NUCL) is a rare form of leishmaniasis described in areas of visceral leishmaniasis (VL) transmission in Central America, including Honduras, Costa Rica, El Salvador, and Nicaragua. *Leishmania (L.) infantum chagasi* is implicated as the aetiological agent that is transmitted by *Lutzomyia longipalpis* sand flies that bite the vertebrate hosts [1]. The patients do not present clinical signs of VL, nor a previous history of visceral diseases. The lesions are characterized by small nonulcerative erythematous papules or erythematous plaques of chronic evolution that are between 1 to 10 mm in diameter and are located in exposed areas of the body, especially the face and extremities, often surrounded by a hypopigmented halo. The main tissue features have been characterized by a granulomatous reaction with a small number of amastigote forms of the parasite [2].

It is important to mention that the identification of *Leishmania* isolates from NUCL lesions from Honduras showed that parasites belong to the *Leishmania donovani* complex by specific monoclonal antibodies, and they were identified as *Leishmania (L.) donovani chagasi* by isoenzyme analysis [1]. Despite NUCL, VL occurs in the same endemic areas in Honduras, and it should be noted that NUCL is the most common form of the clinical presentation of *Leishmania (L.) infantum chagasi* infection; it affects children older than 6 years and young adults more frequently, while VL occurs mainly in children younger than 5 years [1, 3, 4].

Studies have suggested the ability of *Leishmania (L.) infantum chagasi* to cause both clinical forms, while VL and NUCL could be related to the immunological and genetic backgrounds of the host, as well as parasite and sand fly vector characteristics [2–4]. However, little is known about the profile of human infection by *Leishmania (L.) infantum chagasi* in Honduras, especially of the nonulcerated or atypical form. We have observed self-limiting and nonulcerated skin lesions independent of the disease evolution time, characterized by mononuclear inflammatory infiltration composed mainly of lymphocytes, vacuolated macrophages associated with granulomatous reactions, and scarce parasites, suggesting an efficient cellular immune response in the skin of individuals affected by NUCL. However, the persistence of low tissue parasitism may be related to the regulatory immune response responsible for balanced cellular immune responses that prevent the evolution of the lesion size and lead to lasting immunity. Therefore, the aim of the present study was to characterize the immune regulatory response in skin lesions of patients affected by nonulcerated or atypical cutaneous leishmaniasis in order to better understand the pathogenesis of the infection caused by this species of parasite in Central America.

## 2. Material and Methods

### 2.1. Study Area

Two endemic areas of nonulcerated or atypical cutaneous leishmaniasis, Amapala and Orocuina municipalities, located in the southern region of Honduras, were studied. These regions have an average annual temperature of 30°C, with a maximum ranging between 34°C–35°C, a minimum between 25°C–26°C, and an annual humidity of 65% [5].

### 2.2. Casuistry

Twenty skin biopsies from patients with NUCL, without treatment, and with parasitological diagnosis confirmed by scraping of lesions and stained by Giemsa were used. Patients were informed about the research protocol, and those who agreed to participate signed the informed consent form. This work was approved by the Research Ethics Committee of the Master of Infectious and Zoonotic Diseases of the National Autonomous University of Honduras (Protocol number 03-2014) and by the Research Ethics Committee of the Medical School of the University of São Paulo (CAAE: 64223917.1.0000.0065, Protocol: 1.938.092).

### 2.3. Histopathology

The biopsies of skin lesions from patients, defined as nonulcerative, erythematous papules, infiltrative plaques, or nodules, in the presence or absence of hypopigmented halos, were collected using a 3 mm punch under aseptic conditions and under local anaesthesia. These biopsies were immersed in 10% formalin solution buffered with 0.01 M phosphate and processed by the usual histological techniques to obtain the paraffin sections. Paraffin sections stained by haematoxylin-eosin (HE) were observed under an optical microscope, with the goal of characterizing histopathological changes. A semiquantitative comparative analysis of the sections stained by HE was performed according to the adaptation of Ridley and Ridley [6], assigning scores for the intensity of the different characterized processes, where (−) is negative, (+) is discrete, (++) is moderate, and (+++) is intense.

### 2.4. Immunohistochemistry

The in situ regulatory response was assessed by immunohistochemistry using the following markers: anti-CD4 monoclonal antibody and anti-FoxP3, anti-TGF-*β*1, and anti-IL-10 polyclonal antibodies. Hyperimmune serum from a mouse chronically infected with *Leishmania* (*Leishmania*) *amazonensis* was used to confirm tissue parasitism. Histological sections of 4 *μ*m thickness were deparaffinized in xylene for 15 minutes, followed by hydration with a descending series of alcohols; endogenous peroxidase was blocked with 3% hydrogen peroxide solution. Antigen retrieval was conducted using 10 mM citrate buffer at pH 6.0 in a boiling water bath. After this step, primary antibodies were added to the tissues in the following dilutions: anti-*Leishmania* (mouse hyperimmune serum produced in our laboratory, Moreira et al. [7]) diluted at 1 : 2000; anti-CD4 (monoclonal, NCL-L-CD4-1F6, Novocastra) diluted at 1 : 20; anti-FoxP3 (polyclonal, (H-190): SC-28705, Santa Cruz Biotechnology) diluted at 1 : 250; anti-TGF-*β*1 (polyclonal, (V): SC-146, Santa Cruz Biotechnology) diluted at 1 : 100, and anti-IL-10 (polyclonal, ab34843, ABCAM) diluted at 1 : 1000. As a negative control, a solution containing phosphate-buffered saline (PBS) and bovine serum albumin (BSA) with the omission of the primary antibody was used. The slides were incubated in a humidified chamber overnight at 4°C. For all markers, the Novolink kit (RE7280-K—Leica) was used. The chromogenic substrate, DAB + H_2_O_2_ (diaminobenzidine with hydrogen peroxide—K3468—DakoCytomation), was added to the tissue, incubated for 5 minutes, and counterstained with Harris haematoxylin. Finally, the slides were dehydrated in a series of ascending alcohols and mounted with Permount and glass coverslips.

Ten skin samples obtained from healthy individuals undergoing plastic surgery were included as controls.

### 2.5. Quantitative Analysis of Immunostained Cells

Images were obtained using an optical microscope coupled to the microcomputer, and quantification of immunostained cells was performed using AxioVision 4.8.2 software (Zeiss, San Diego, CA, USA). Ten microscopic fields of each histological section for different markers were imaged by a 40x objective, and the cells immunostained in brown were quantified. The cellular density (number of cells per square millimetre) was determined by the ratio of the immunolabelled cells to the area of each image.

### 2.6. Statistical Analysis

For the statistical analysis of the results, GraphPad Prism 5.0 software was used and to analyse the difference between the different groups, the *t* test was performed for the Gaussian distribution data, and the Mann-Whitney test was used for the non-Gaussian distribution data.

## 3. Results

### 3.1. Histopathological Features

**Figure 1:**
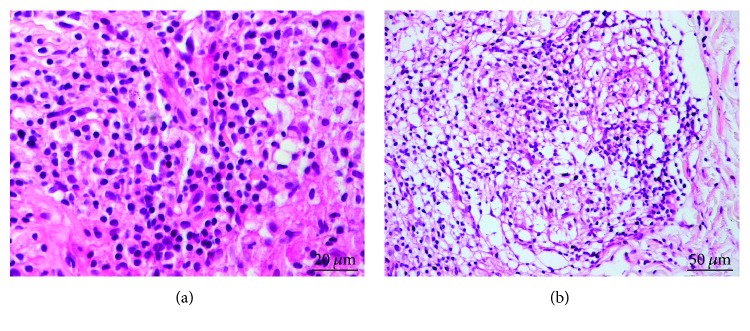
Histological section of a skin biopsy from a patient affected by nonulcerated cutaneous leishmaniasis showing intense mononuclear inflammatory infiltration in the dermis (a) and epithelioid granuloma (b).

In the histopathological analysis, the skin lesions were characterized by mononuclear inflammatory infiltration in the dermis, composed predominantly of lymphocytes, followed by vacuolated macrophages and a few plasma cells. The intensity of the inflammatory infiltration varied from discrete to intense, but in both, the parasitism was discrete. Despite the direct parasitological exam, the presence of the amastigote form of *Leishmania* was in 100% (20/20) of the cases, while histological sections stained by immunohistochemistry evidenced amastigote forms of parasites in only 55% (11/20) of the cases. Granulomas were present in 60% (12/20) of the cases and were associated with moderate to intense inflammation (Figure 1).

### 3.2. Immunohistochemical Analysis

**Figure 2:**
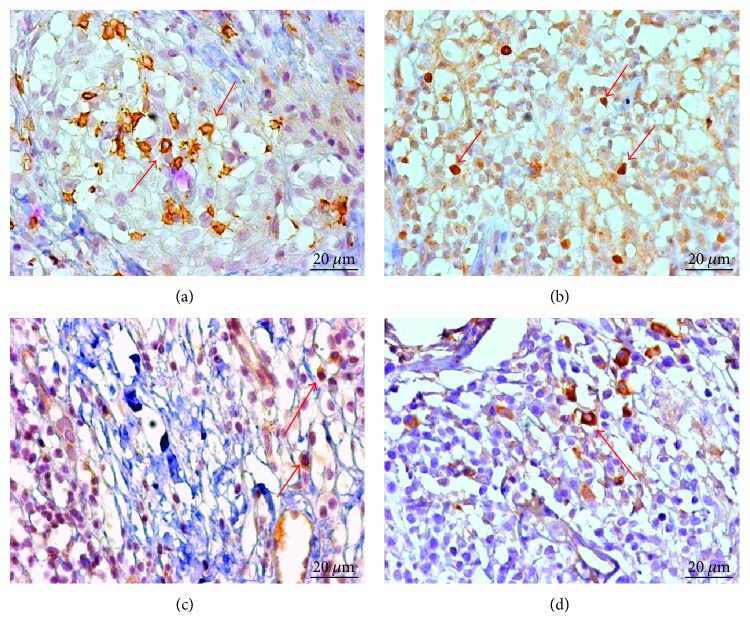
Immunohistochemistry of the skin of patients with nonulcerated or atypical cutaneous leishmaniasis evidenced in brown colour for CD4^+^ T lymphocytes (a); FoxP3^+^ cells (b); IL-10^+^ cells (c), and TGF-*β*^+^ cells (d) (×400). The red arrows signal immunostained cells for the different markers.

The skin lesions of NUCL patients showed the presence of CD4^+^ T, FoxP3^+^ lymphocytes, TGF-*β*^+^, and IL-10^+^ cells, which were evidenced by the immunohistochemical reaction (Figure 2).

**Figure 3:**
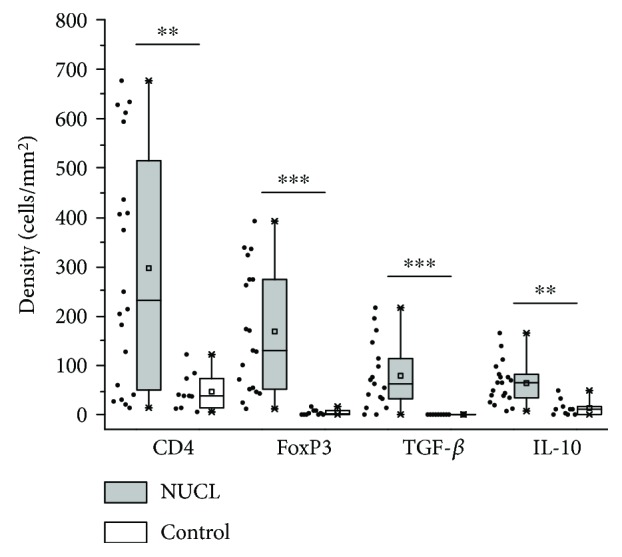
A dot plot showing the distribution and a box plot showing the median, mean, quartile, maximum, and minimum values for the number of positive cells per square millimetre for CD4, FoxP3, TGF-*β*, and IL-10 markers in the skin biopsies of nonulcerated cutaneous leishmaniasis (grey) and healthy individuals (white). ^∗∗^*p* < 0.01; ^∗∗∗^*p* < 0.001 of cellular density between NUCL and healthy controls.

The quantitative morphometric analysis of the skin lesions of patients affected by nonulcerated or atypical cutaneous leishmaniasis showed that the cellular density (mean ± standard error) of CD4^+^ T lymphocytes was 296.60 ± 53.47, that of FoxP3^+^ cells was 168.40 ± 28.71, that of TGF-*β*^+^ cells was 78.63 ± 16.54, and that of IL-10^+^ cells was 63.72 ± 9.70 cells/mm^2^. However, in skin from healthy individuals, the number of cells/mm^2^ was 46.25 ± 11.55 for CD4^+^ T lymphocytes, 3.79 ± 1.72 for FoxP3^+^ cells, 0.11 ± 0.11 for TGF-*β*^+^ cells, and 13.26 ± 5.05 for IL-10^+^ cells. The densities of CD4^+^ T cells and IL-10^+^ cells were higher in NUCL patients when compared to healthy skin (*p* < 0.01), and the densities of FoxP3^+^ cells and TGF-*β*^+^ cells were higher in NUCL patients compared to healthy skin (*p* < 0.001) (Figure 3).

The ratio between regulatory FoxP3^+^ and effector CD4^+^ T cells, as well as the ratio between positive cytokines (TGF-*β*^+^ and IL-10^+^) and FoxP3^+^ and CD4^+^ T cells in NUCL and normal skin, was assessed in order to better evaluate the participation of regulatory cells in the cutaneous inflammation caused by atypical cutaneous leishmaniasis. The ratio of FoxP3 : CD4 was six times higher in NUCL (0.568) than in healthy skin (0.082), the ratio of TGF-*β* : CD4 was one hundred and thirty-two times higher in NUCL than in healthy skin, and the ratio of TGF-*β* : FoxP3 was fifteen times higher in NUCL (0.260 and 0.467, resp.) than in healthy skin (0.002 and 0.029, resp.). Already, the ratio of IL-10 : CD4 was similar between NUCL and healthy skin, at 0.3 times higher in healthy skin (0.286) than in NUCL (0.215), and the ratio of IL-10 : FoxP3 was eight times higher in healthy skin (3.500) than in NUCL (0.378).

**Figure 4:**
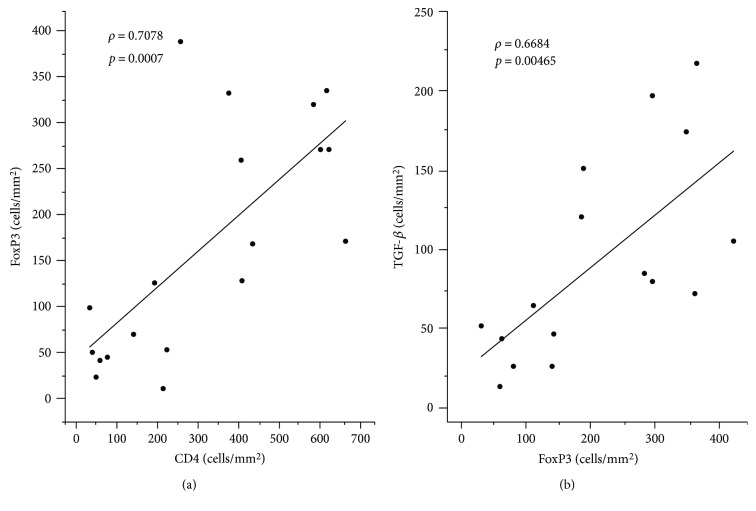
Graphic of dispersion showing a positive and strong correlation between the cellular density of CD4^+^ cells and FoxP3^+^ cells (a) and a positive and moderate correlation between FoxP3^+^ cells and TGF-*β*^+^ cells (b). The value of *ρ* is the Pearson correlation coefficient, and *p* is the *p* value.

A positive and strong correlation was observed between the density of CD4^+^ T cells and FoxP3^+^ (*ρ* = 0.7078, *p* = 0.0007), and a positive and moderate correlation was detected between FoxP3^+^ and TGF-*β*^+^ cell density (*ρ* = 0.6868, *p* = 0.00465); however, the density of IL-10^+^ cells did not show correlation with any other markers (Figure 4).

## 4. Discussion

Nonulcerated or atypical cutaneous leishmaniasis is a rare clinical form of infection caused by *Leishmania (L.) infantum chagasi*, and interestingly, it has been described only in Central America. In Honduras, infection caused by *Leishmania (L.) infantum chagasi* is restricted to the southern region of the country, where cases of VL and NUCL occur in the same geographic area [1]. It is an intriguing fact that since the first cases of NUCL have been described in the country, a reduction in the number of cases of the visceral form of the disease has been noted, accompanied by an increase in cases of the cutaneous form, suggesting an efficient adaptation of the pathogen to the host that leads to a balanced parasite-host relationship.

In our study, the main histopathological changes observed in skin lesions of patients with NUCL were characterized by mononuclear inflammatory infiltration in the dermis formed by lymphocytes and macrophages of variable intensity and associated with the formation of epithelioid granulomas. The presence of a granulomatous reaction was associated with an inflammatory infiltration ranging from moderate to severe, mainly diffuse, with evidence of giant cells and focal necrosis in some cases. Despite very few parasites being observed in 100% of cases by direct parasitological exam, suggestive forms of the parasite were observed in only 55% of the cases in histological sections stained by HE or immunohistochemistry. These histopathological aspects differ from those that have been described in the Old World for cutaneous lesions caused by other viscerotropic species, such as *Leishmania* (*L*.) *donovani* and *Leishmania* (*L*.) *infantum* [8, 9]. This reinforces the role of the parasite species in determining the clinical and immune-histopathological aspects of the infection [10].

A previous study from our group on skin lesions of atypical cutaneous leishmaniasis showed self-limiting and nonulcerated skin lesions independent of the time of evolution, which were characterized by the evidence of a high density of CD8^+^ T lymphocytes and IFN-*γ*^+^ cells, added to the presence of iNOS^+^ macrophages that are rarely parasitized [11]; these results suggested that those patients had efficient cellular immune responses in the skin. However, the persistence of low tissue parasitism could be associated with a regulatory immune response that leads to a balanced cellular immune response [12].

Regulatory T lymphocytes represent a subpopulation of T lymphocytes characterized phenotypically by CD4^+^ CD25^+^ cells expressing transcription factor forkhead box P3 (FoxP3), essential for the control of excessive immune response against microorganisms or self-antigens. Regulatory T cells (T_reg_) act in conjunction with effector T cells on the modulation of the cellular immune response [13–16]. Therefore, the role of T_reg_ cells is mediated by the secretion of regulatory cytokines, such as IL-10 and TGF-*β*, which directly affect the activity of effector T cells and antigen-presenting cells [12–14]. The production of these cytokines at the site of infection can compromise the proper proliferation of effector T cells and the production of proinflammatory cytokines, inhibiting full parasite elimination [12, 17].

Considering the morphology of lymphocytes, we estimated that approximately 10% of the lymphocytes were T_reg_ cells, characterized by FoxP3^+^ cells in our study. In addition, a strong and positive correlation was observed between the number of CD4^+^ T cells and the number of FoxP3^+^ cells (*p* = 0.0007), and the ratio of FoxP3 : CD4 was six times higher in NUCL than in healthy skin, suggesting that a significant part of the CD4^+^ T lymphocyte population was T_reg_ cells in the inflammatory infiltration in the skin lesions caused by NUCL [18].

Moreover, there was a positive and moderate correlation between the density of FoxP3^+^ cells and the density of TGF-*β*^+^ cells (*p* = 0.00465) but not between FoxP3^+^ and IL-10^+^ cells (*p* = 0.53585). Additionally, the ratios of TGF-*β* : CD4 and TGF-*β* : FoxP3 were higher in NUCL than in healthy skin, which did not occur with IL-10. These data suggest that in NUCL, the T_reg_ cells could regulate an effector cellular immune response, mainly through the production of TGF-*β*, a cytokine that depends on the environment and concentration at which it is produced to present proinflammatory or anti-inflammatory properties [19–22]. Previously, it has been shown that both T_reg_ and T effector cells are present in chronic leishmaniasis, suggesting that the persistence of *Leishmania* at the site of infection is due to the activity of T_reg_ cells, although it has been believed that the low number of parasites at the site of infection is important to produce long-lasting and protective immunity [12, 23]. Thus, these cells can control the balance of the cellular immune response established between the pathogen and its host, mediating an equilibrium that may be mutually beneficial. An imbalance in this cellular subtype may promote lesion progression and change in the immune cellular response, since CD4^+^ CD25^+^ IL-10^+^ TGF-*β*^+^ cells could be involved in the modulation of the effector immune response in skin lesions induced by *Leishmania* spp. [12, 17, 24]. In addition, it was demonstrated that the chronicity of the skin lesions caused by *Leishmania guyanensis* is associated with the immunosuppression due to the presence of T_reg_ cells, suggesting that T_reg_ cells could play a role in the downregulation of *Leishmania*-specific immune responses [25, 26].

The lack of correlation between FoxP3^+^ and IL-10^+^ cells suggests that other regulatory cells could be the source of IL-10, since it has been described that IL-10-producing CD25^−^ Foxp3^−^ T cells are involved in the pathogenesis of visceral leishmaniasis [27]. Moreover, skin lesions of patients affected by cutaneous leishmaniasis caused by *L. (V.) braziliensis* showed a stronger correlation between IL-10 expression and proinflammatory cytokines such as IFN-*γ*, IL-27, and IL-21, rather than with FoxP3^+^ cells [28].

Taken together, the data obtained in this study suggest that CD4^+^ T lymphocytes and FoxP3^+^ T regulatory cells, as well as TGF-*β*^+^ and IL-10^+^ cells, although discrete, play an important role in the immunopathogenesis of nonulcerated or atypical cutaneous leishmaniasis. These elements could modulate the balance in the cellular immune response, resulting in the maintenance of low tissue parasitism necessary for protective immunity that prevents the evolution of the lesion size.
